# The Role of Social Integration in Chronic Disease Prevalences Among the Internal Migrant Populations in China: Evidence from a National Survey

**DOI:** 10.3390/healthcare13010069

**Published:** 2025-01-02

**Authors:** Xiao Yang, Yixuan Zhang, Siyu Zou, Yihang Chen, Ziqing Cai, Ying Zhu, Kun Tang

**Affiliations:** 1 Department of Health Policy and Management, Johns Hopkins Bloomberg School of Public Health, Baltimore, MD 21205, USA; xyang97@jh.edu; 2 Vanke School of Public Health, Tsinghua University, Beijing 100084, China; 3 Department of Epidemiology, Johns Hopkins Bloomberg School of Public Health, Baltimore, MD 21205, USA; 4 School of Health Humanities, Peking University Health Science Center, Beijing 100191, China; 5 School of Law and Humanities, China University of Mining and Technology-Beijing, Beijing 100083, China; 6 Xuanwu Hospital of Capital Medical University, Beijing 100037, China

**Keywords:** internal migrants, social integration, China, chronic disease, health status, hypertension, diabetes

## Abstract

**Background**: China has the world’s largest internal migrant population, yet chronic disease prevalence among this group remains largely overlooked. The integration of the internal migrant population into the local society may affect their noncommunicable disease prevalences and become a challenge for the public health system. This study aimed to explore the association between the social integration of China’s internal migrant population and the prevalences of chronic diseases, including hypertension and diabetes. **Methods**: This study used data from the 2017 China Migration Dynamic Survey. Social integration status was assessed using an 8-item Likert scale and categorized into four quartiles, with higher points indicating higher levels of social integration. Multivariate logistic regression was conducted to examine the association between social integration level and the prevalences of hypertension, diabetes and combined chronic diseases. Disaggregated analysis was performed to explore the potential effect modification by age, sex, income, and migration duration. **Results**: A medium level of social integration was associated with lower prevalences of chronic diseases, while the lowest and highest levels of social integration were both associated with enhanced prevalence. Further disaggregation demonstrated the relationship between social integration and chronic disease prevalences were modified by various factors, including age, sex, income, and migration duration. **Conclusions**: This study demonstrated that both the lowest and highest levels of social integration can significantly affect chronic disease outcomes of China’s internal migrants. These findings emphasize the necessity to formulate tailored public health policies to effectively prevent and manage chronic diseases among the internal migrant population in China.

## 1. Introduction

China’s internal migrant population, also referred to as a floating population, are individuals who have relocated from their registered household residences to more developed regions within the country [1,2]. This movement is primarily driven by the pursuit of urbanized lifestyles, better employment opportunities, and improved economic conditions [3,4]. Over the past few decades, due to China’s rapid economic growth and urbanization progress, the size of the internal migrant population has expanded significantly. Unlike external migrants who constitute a relatively small proportion of the total population due to strict policy controls on migration into China, internal migrants have unrestricted mobility within the country’s geographical boundaries, which contributes to their significantly larger population size [5,6]. Recent census data indicate that China’s internal migrant population has exceeded 375 million, accounting for 27% of the total population, with an average age of 43 years, demonstrating a rising trend in both size and age [7,8]. Consequently, the health status of this community is critical to China’s health prosperity.

Despite their large numbers, members of the internal migrants are often considered non-permanent residents in their destination areas under China’s household registration system (the “Hukou” system) [1]. As a result, substantial disparities exist in the public health services available to them. Compared to the local residents, these internal migrants face lower health awareness and restricted access to healthcare services, primarily due to social inequalities [9,10,11,12,13,14,15]. Additionally, their limited protection under healthcare insurance further exacerbates their vulnerability, contributing to significant public health challenges, such as the management of chronic diseases [14,16,17,18,19,20].

The “healthy migrant effect” suggests that social integration can be a key determinant of health outcomes for migrants [21,22,23]. The degree to which the migrants integrate into the local society may influence their living habits, dietary patterns, health awareness, and service utilization. However, research indicates that the social integration among China’s internal migrant population remains at a relatively low level [24]. Social integration for this group tends to only meet their basic needs, without sufficient mechanisms to support deeper integration into the local communities. Studies show that higher levels of social integration are associated with improved mental health, better health awareness and healthcare service access among the internal migrant population in China [25,26,27]. Yet, evidence regarding the impact of social integration on chronic diseases, such as hypertension and diabetes, remains limited.

Hypertension and diabetes are two major non-communicable diseases (NCDs) affecting the Chinese adult population, with serious implications for their quality of life and life expectancy [28,29,30]. The complications arising from these conditions, such as cardiovascular and renal issues, are leading causes of mortality in China and impose a heavy burden on the individuals, families, and the national healthcare system [30,31,32]. Research has shown that 15% of the internal migrant population suffers from hypertension and 5% from diabetes [33,34]. Although these rates are lower than those of the general population, the unique challenges faced by the internal migrant population—such as poor disease management due to their transient lifestyle and limited healthcare access in the inflow areas—amplify the health risks they face. These chronic conditions present a substantial challenge not only to individuals but also to the broader healthcare system, with insufficient supporting evidence and policy measures in place to address this growing issue. This study aims to investigate the association between social integration and chronic disease prevalences of the internal migrant population in China, focusing on hypertension and diabetes.

## 2. Materials and Methods

### 2.1. Study Design and Participants

This study was a secondary analysis of data from the 2017 China Migrant Dynamic Survey (CMDS) database, a cross-sectional survey implemented by the National Health Commission of China, aiming to investigate the internal migrant population across 32 provincial-level administrative regions in mainland China. Three indicators, including unmarried rate and employment rate among the working-age population, and the proportion of internal migrants intending to reside locally, were used by the 2017 CMDS for the estimation of the sample size, with a relative error controlled within 3% at a 95% confidence level. At the provincial level, the relative error is controlled within a range of 5% to 15%, which results in a total sample size of approximately 170,000 individuals who migrated internally. Using a multistage, stratified, probability proportional to size (PPS) sampling method, a total of 169,989 internal migrants aged 15 or above were recruited. The primary sampling units (PSUs) consisted of 1290 township-level units (townships, towns, streets). The secondary sampling units (SSUs) consisted of villages or neighborhood committees. In the third stage, 169,989 internal migrants who had been living in an SSU for more than one month and did not hold local Hukou were recruited. The survey questionnaire collected data on demographic and socio-economic characteristics, migrant status, health awareness, access to health and public service, social integration status, and key disease conditions of China’s internal migrant population. The details of the sampling method have been described in previous publications [35,36].

**Figure A1:**
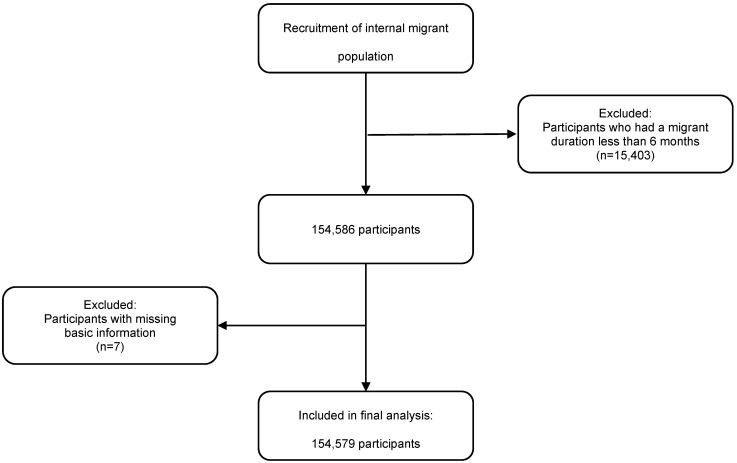
Flow diagram of participant inclusion.

According to China National Bureau of Statistics, internal migrants refer to the population who separated from their households for 6 months and above [37]. Therefore, 15,403 respondents who resided in an SSU for less than 6 months and 7 participants for missing values were excluded from this study and 154,579 participants were included in the final analyses (Figure A1).

### 2.2. Measures

#### 2.2.1. Independent Variable

Social integration was measured by an 8-item Likert scale to report the integration status of the internal migrants, with each item scored from 1 to 4 points (Table A1). Five of these items were positive statements (totally disagree = 1, disagree = 2, agree = 3, totally agree = 4), and the other three were reverse statements (totally disagree = 4, disagree = 3, agree = 2, totally agree = 1). The validity and reliability of the scale have been validated previously [38]. Scores for the 8 items were then summed to obtain a scale ranging from 8 to 32 with higher scores indicating better social integration [39]. The social integration index was further classified into four quartiles (Q1: 8–22; Q2: 23–24; Q3: 25–27; and Q4: 28–32).

#### 2.2.2. Dependent Variables

Hypertension and diabetes were identified by self-reported diagnosis. Participant was asked “Has a doctor ever told you that you had Hypertension or Diabetes?” Those who answered “Yes” to the questions were classified with self-reported hypertension or diabetes, respectively. Participants who reported either or both of these two conditions were classified as having combined chronic diseases.

#### 2.2.3. Covariates

Demographic (age, sex, ethnicity, marital status, location of hometown), socioeconomic (monthly income, education, medical insurance, social insurance) and migrant status (migration duration, migration range, and participation in social activity) were included in the analysis (Table A2).

### 2.3. Statistical Analysis

Descriptive analyses were performed to calculate the frequency distribution and percentages for categorical variables. Continuous variables were summarized using median and interquartile range (IQR). Differences in continuous variables across quartiles of the social integration index were assessed using *t*-test. For categorical variables, Pearson’s chi-squared test was applied to evaluate variations in prevalence across social integration index quartiles. Multivariable logistic regression analysis was conducted to examine the association between social integration and three sets of chronic disease prevalences, including hypertension (yes/no), diabetes (yes/no), and chronic diseases (yes/no). The models were adjusted for the covariates mentioned above.

In further disaggregated analysis, the potential modification effect of age, sex, income, and migration duration were examined in the logistic regression model. Specifically, disaggregated analyses were conducted by each variable, including age (<35 years, ≥35 years), sex (male, female), income (< CNY 6000, ≥ CNY 6000, and migration duration (<10 years, ≥10 years). The age and income strata were determined by the median values of the sample, while the disaggregation for migration duration was based on the cumulative burden of chronic diseases. A duration of 10 years is applied according to evidence from previous literature [40]. The model was adjusted for covariates. When performing disaggregated analysis based on age, sex, income, and migration duration, the respective variable was excluded.

Factors associated with chronic disease prevalences were reported as adjusted odds ratios (AORs) or coefficient and their 95% confidence intervals (CIs). Associations were determined statistically significant if *p*-values were less than 0.05 (two-sided). All analyses were performed using Stata 17 MP (StataCorp., College Station, TX, USA).

## 3. Results

### 3.1. Baseline Characteristics

Table 1 presents the baseline characteristics of 154,579 participants, grouped by social integration index quartiles. The distribution of age and sex was relatively balanced, with approximately half of the participants under 35 years old (79,102, 51%) and half being male (79,576, 51%). Nearly two-thirds of the participants’ education level was middle school and below (93,537, 60%). A significant majority (135,345, 88%) have migrated from rural areas. The overall prevalence of hypertension, diabetes, and combined chronic diseases were 5%, 1%, and 6%, respectively. The median social integration score was 24 (IQR: 23.0–27.0), with a median score of 22.0 (IQR 20.0–22.0), 23.0 (IQR 23.0–23.0) for the second quartile, 25.0 (IQR 24.0–26.0) for the third quartile, and 29.0 (IQR 28.0–30.0) for the fourth quartile, respectively. Participants with higher social integration scores tended to have higher monthly incomes, higher education levels, were more likely to be currently married, had longer migration durations, narrower migration ranges, and better access to medical insurance, social insurance, and more engagement in social activities.

### 3.2. Association Between Social Integration Quartile and Chronic Disease Outcomes

The AORs and 95% CIs for hypertension, diabetes, and combined chronic diseases were presented in Table 2. Participants in higher social integration quartiles (Q2, Q3, and Q4) exhibited a varying likelihood of chronic diseases when compared to those in the lowest quartile (Q1). Individuals in Q2 were 11% less likely (AOR 0.89, 95% CI: 0.87–0.90) and 4% less likely in Q3 (AOR 0.96, 95% CI: 0.95–0.97), respectively, to report hypertension; whereas individuals in Q4 were 8% more likely to report hypertension (AOR 1.08, 95% CI: 1.03–1.13). As for diabetes, the association followed a similar trend, with no significant association in Q2 (AOR 0.98, 95% CI: 0.93–1.03) and a 25% higher likelihood of having hypertension in Q4 (AOR 1.23, 95% CI: 1.15–1.32). For combined chronic disease prevalences, participants in Q2 and Q3 had lower odds ratios (AOR 0.89, 95% CI: 0.88–0.90 and AOR 0.96, 95% CI: 0.94–0.99) compared to an increased odds ratio in Q4 (AOR 1.09, 95% CI: 1.03–1.14).

### 3.3. Effect Modification by Age, Sex, Income, and Migration Duration

**Figure 1:**
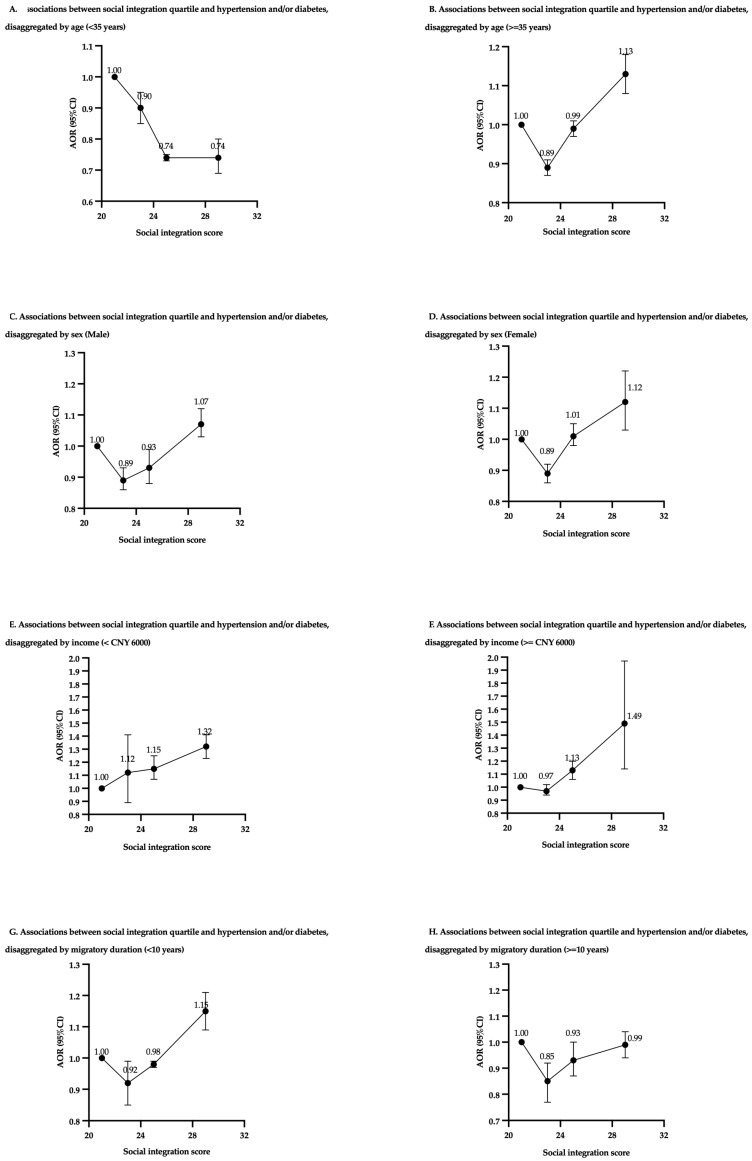
Association between social integration and hypertension and/or diabetes, performed by logistic regression analysis and disaggregated by each variable including age, sex, income, and migrant duration. AOR, adjusted odds ratio; CI, confidence interval. The model has been adjusted for age, sex, monthly income, education, ethnicity, marital status, medical insurance, hometown type (rural, suburban, urban), migration duration, migration range, social insurance, and participation in social activity. When performing disaggregated analysis based on age, sex, income, and migratory duration, the respective variable was excluded from the model adjustments.

The association between social integration and the prevalence of hypertension, diabetes, and combined chronic diseases was further investigated through disaggregated analyses by each variable, including age, sex, income, and migration duration (Figure 1 and Table A3).

#### 3.3.1. Age

In the disaggregated analysis by age, the association between social integration and combined chronic diseases differed significantly between participants under 35 years and those 35 years and above (Figure 1A,B). For participants under 35 years, the trend indicates higher social integration was associated with a lower likelihood of chronic diseases, as demonstrated by a decreasing AOR across social integration quartiles (Q2: AOR 0.90, 95% CI: 0.85–0.95; Q3: AOR 0.74, 95% CI: 0.73–0.75; Q4: AOR 0.74, 95% CI: 0.70–0.78). In contrast, the association for participants aged 35 years and above showed a U-shaped pattern, where the likelihood of having chronic diseases reached the lowest level at Q3 (AOR 0.89, 95% CI: 0.87–0.91), while Q4 displayed a thirteen percent increased likelihood compared to Q1 (AOR 1.13, 95% CI: 1.08–1.18).

#### 3.3.2. Sex

For males, Q2 was associated with a reduced proportion of reporting hypertension and/or diabetes (AOR 0.89, 95% CI: 0.86–0.93), while the likelihood significantly increased by 16% in Q4 (AOR 1.16, 95% CI: 1.02–1.31) (Figure 1C). A similar U-shaped pattern was observed for females, where Q2 had the lowest odds ratio (AOR 0.81, 95% CI: 0.72–0.90), and the odds ratio in Q4 elevated marginally compared to Q1 (AOR 1.02, 95% CI: 0.91–1.14) (Figure 1D).

#### 3.3.3. Income

For participants with incomes below CNY 6000 (Figure 1E), there was a linear decrease in the odds ratios of chronic diseases with the level of social integration increased. The highest quartile of social integration was associated with a 26% lower likelihood of having hypertension and/or diabetes (AOR 0.74, 95% CI: 0.67–0.80). Conversely, for participants with incomes ≥ CNY 6000 (Figure 1F), the relationship was less significant, with little variation in the odds ratios of chronic diseases across social integration quartiles (AOR 1.07, 95% CI: 0.90–1.12).

#### 3.3.4. Migration Duration

Disaggregation by migration duration highlighted medium social integration offered a protective effect regardless of the length of time participants have migrated (Figure 1G,H). Among the population who have migrated for less than 10 years (Figure 1G), a U-shaped pattern was observed, where both Q2 and Q3 exhibited lower odds ratio (Q2: AOR 0.92, 95% CI: 0.85–0.99; Q3: AOR 0.98, 95% CI: 0.97–0.99), while the highest quartile was associated with a 49% increase in chronic disease prevalence (AOR 1.49, 95% CI: 1.14–1.97). For those with a migration duration of 10 years and above (Figure 1H), a similar pattern was identified in Q2 and Q3 (Q2: AOR 0.85, 95% CI: 0.77–0.92; Q3: AOR 0.93, 95% CI: 0.87–1.00), while Q4 was not associated with a significantly increased level of chronic disease prevalence (AOR 0.99, 95% CI: 0.94–1.04).

## 4. Discussion

This study investigated the association between the level of social integration and the prevalences of hypertension, diabetes, and combined chronic diseases among the internal migrant populations in China. Interestingly, the results indicated that the medium level of social integration was associated with a reduced likelihood of reporting chronic conditions, while those with the highest or lowest levels of social integration exhibited an increased likelihood of having hypertension, diabetes, or either disease. Additionally, further analysis revealed effect modifications in the relationship between social integration and the prevalences of chronic disease by various factors, including age, sex, income, and duration of migration.

Overall, our research highlighted that internal migrant populations with the highest level of social integration generally exhibited the greatest likelihood of developing hypertension, diabetes, or both conditions. In contrast, those with medium levels of social integration had a relatively lower burden of chronic illnesses. This finding aligns with the “healthy migrant effect” observed in international migrant populations [41]. A study of Chinese immigrants in Italy found that immersing into local culture was linked to a higher prevalence of hypertension and type 2 diabetes [42]. A similar study on Chinese American immigrants showed better cultural adaptation was significantly associated with elevated blood pressure [43]. Additional studies demonstrated that greater acculturation among immigrants increased the likelihood of hypertension, diabetes, and the figure could be doubled among certain immigrant communities [44,45,46]. Similar to international migrants, the internal migrant population in China typically moves from less developed to more developed regions, pursuing better living conditions, higher incomes, and less physically demanding jobs [47,48,49]. These changes often lead to altered dietary habits with increased calorie intake, reduced physical activity, and more sedentary lifestyles, which can increase the probability of having chronic diseases [50,51,52]. Internal migrants with the lowest levels of social integration showed higher rates of chronic diseases, likely due to the stress of adapting to a new environment. Conversely, those with the highest levels of integration had the greatest burden of hypertension and diabetes, possibly because they had better access to healthcare and were more likely to be diagnosed. However, the pressure to fit in and maintain larger social networks may also cause stress, negatively impacting their health [53]. As several studies suggested, the effects of an individual’s wider social network on health are barely comparable to that of intimate personal relationships, which is much more beneficial to health [54,55,56].

Further analysis revealed different trends in the relationship between social integration and chronic disease prevalences across different age groups within the internal migrant populations. Among respondents under 35 years old, higher levels of social integration were consistently associated with a lower prevalence of chronic diseases. However, for those aged 35 and above, social integration exhibited a U-shaped relationship with chronic disease prevalence, with the highest level of integration corresponding to the greatest prevalence. Younger internal migrants are generally more proactive in adapting to their new environment [57,58]. Compared to the older internal migrants, increased social integration means that they may have greater access to services and are able to seek more actively for health education resources [59,60,61]. In contrast, older internal migrants in China often relocate to assist their adult children who reside in the inflow region, or they are the first-generation internal migrants themselves [49,62]. They need to provide more physical or financial support to their families, often at the expense of their own time and money, which can lead to a passive social integration pattern. Alternatively, when the elderly internal migrants need assistance to access to healthcare services, their children are often unable to provide companionship and care because they are in school or work, so that the senior internal migrants cannot receive timely and appropriate diagnosis and treatment due to lack of intergenerational support [63,64]. In a typical society in China, where social and community support is not as well established as traditional family care, higher levels of social integration alone could not provide close support like family members, and may impose additional stress, thereby increasing the likelihood of having chronic diseases in this group [65,66,67].

The relationship between social integration and chronic disease prevalences in male and female internal migrant populations both follow a U-shaped pattern, with a medium level of social integration offering stress-buffering benefits for both. However, women experience a steeper curve and a narrower range of the protective effect from social integration. This may be a result of different integration patterns between sexes [68,69]. Divney et al. discovered that female immigrants with higher acculturation had a 50% higher proportion of having hypertension compared to those with lower acculturation, while the likelihood for male immigrants increased 15% [45]. Similarly, Modesti et al. found that acculturation nearly doubled the prevalence of hypertension and diabetes among female Chinese immigrants in Italy, with no significant impact on men [42]. A study of immigrant women from Mexico and the Middle East has shown similar results [70,71]. These disparities may arise from the traditional role for different sexes, which places greater family care responsibilities on women, as well as barriers to social integration, such as gender discrimination. At the migration destination, male migrants tend to build broader external social networks, often through work, while women typically maintain smaller, family-centered networks [68,72,73,74,75]. As women reach higher levels of social integration, the exhaustive effort to balance family and social responsibilities may exert excessive burden and compromise their health [76,77,78].

Meanwhile, irrespective of the internal migrant population’s income level, higher levels of social integration are significantly associated with a higher likelihood of developing chronic diseases. This may be attributed to the income disparity between the migrant community and local residents, as the study found socioeconomic inequality negatively influences the health of internal migrants with equivalent levels of social integration compared to local residents [25]. The development and maintenance of social networks is one of the strategies used by the migrant population to increase income at the inflow region; however, they often have to endure the down-side of frequent social interactions, such as picking up unhealthy social behaviors including smoking, drinking, and intaking high-calorie food which can contribute to chronic disease development [25,79,80,81,82,83,84].

Finally, in the disaggregated analysis by migration duration, better social integration was associated with higher prevalences of chronic diseases among individuals with a migration duration < 10 years. In comparison, this trend was not significant among those who have migrated ≥ 10 years. However, in the linear regression analysis, it was found that internal migrants who have migrated ≥ 10 years have a significantly higher likelihood of having chronic diseases than those who have migrated < 10 years. We believe that this difference is due to the use of relative comparisons rather than comparisons of absolute values in the disaggregated analysis. Although no significant increase in the prevalences of chronic diseases was observed in the group whose have migrated ≥ 10 years, this may be attributed to the fact that other important factors, such as better social welfare and access to healthcare services, which may exert stronger effects on migrants of ≥10 years, could potentially undermine such an association.

Meanwhile, our findings regarding the association between social integration and chronic disease development disaggregated by migration duration are not in contradiction with the “healthy migrant effect” [41]. However, previous migrant studies that investigated the “healthy migrant effect” were often limited by relying solely on time as a measure of migrants’ social integration [43,44,45,46,85,86,87]. To address this limitation, this study employed a broader range of indicators, including participants’ subjective sense of integration and perceptions of social acceptance. Additionally, we conducted disaggregated analyses to examine how different subgroups and their social integration status may influence the likelihood of developing chronic diseases. While these indicators did not show a significant impact among the long-term internal migrants, future studies could benefit from incorporating more comprehensive dimensions, such as economic integration, social support, self-identity, or using standardized scales to comprehensively evaluate the relationship between social integration and health outcomes [55,88,89,90,91].

Admittedly, this study also has several limitations. First, this study focuses on internal migrants in China. Given the unique characteristics of China’s household registration system and the relatively smaller differences in language, culture, race, and political environment among internal migrants compared to international migrants, the findings of this study may not be fully generalizable to international migrant populations. Moreover, as a cross-sectional analysis, it cannot establish causality between social integration and chronic disease prevalences. In addition, reverse causality may also occur due to the cross-sectional study design, as the internal migrants’ physical and mental health may affect their social participation and long-term settlement intention in the destination region, which are key aspects for social integration [92]. While our study showed significant associations between social integration and chronic disease prevalences, the direction of the causality between social integration and health among the internal migrants shall be further explored by cohort studies. The social integration scale reflects subjective perceptions rather than actual community acceptance, and the reliance on self-reported data for hypertension and diabetes lacks objective measures like blood pressure and glycated hemoglobin levels. Moreover, the 2017 CMDS database contains the most recent data available for our study, which may present certain limitations in capturing the changing demographics of the internal migrant populations. However, as the Hukou system remains the decisive factor differentiating the internal migrants from local residents and has undergone no major reforms since 2014, we believe the large, representative sample from the 2017 CMDS database still holds significant relevance and provides valuable guidance to the understanding of the current situation. When updated data become available, future research should adopt longitudinal designs and incorporate more objective health and social integration measures to explore the changing dynamics and to establish their relationship with chronic disease prevalences.

## 5. Conclusions

This is one of the few studies, to our knowledge, that examined the relationship between social integration and chronic disease prevalences among China’s internal migrant population. The findings suggest that both the highest and lowest levels of social integration were associated with increased prevalences of chronic diseases. These insights provide valuable guidance for policymakers and healthcare providers in designing targeted social integration strategies and chronic disease prevention programs for different internal migrant populations in China.

## Figures and Tables

**Table 1:** Baseline characteristics.

Characteristics	Total N = 154,579	Social Integration Quartile				*p*-Value ^1^
		Q1 (8–22)	Q2 (23–24)	Q3 (25–27)	Q4 (28–32)	
		N = 34,778	N = 22,515	N = 58,790	N = 38,496	
**Social integration score ^2^**	24.00 (23.00, 27.00)	21.00 (20.00, 22.00)	23.00 (23.00, 23.00)	25.00 (24.00, 26.00)	29.00 (28.00, 30.00)	<0.001
**Age**						<0.001
<35	77,222 (49.96%)	17,289 (49.71%)	11,677 (51.86%)	29,764 (50.63%)	18,492 (48.04%)	
≥35	77,357 (50.04%)	17,489 (50.29%)	10,838 (48.14%)	29,026 (49.37%)	20,004 (51.96%)	
**Sex**						<0.001
Male	79,576 (51.48%)	18,173 (52.25%)	11,708 (52.00%)	30,168 (51.31%)	19,527 (50.72%)	
Female	75,003 (48.52%)	16,605 (47.75%)	10,807 (48.00%)	28,622 (48.69%)	18,969 (49.28%)	
**Income**						<0.001
<6000	72,959 (47.20%)	17,309 (49.77%)	10,886 (48.35%)	27,390 (46.59%)	17,374 (45.13%)	
≥6000	81,620 (52.80%)	17,469 (50.23%)	11,629 (51.65%)	31,400 (53.41%)	21,122 (54.87%)	
**Educational achievement**						<0.001
Middle school/Primary school/No formal school	93,537 (60.51%)	24,666 (70.92%)	14,116 (62.70%)	34,456 (58.61%)	20,299 (52.73%)	
Secondary school	33,782 (21.85%)	6499 (18.69%)	4942 (21.95%)	13,329 (22.67%)	9012 (23.41%)	
College and above	27,260 (17.64%)	3613 (10.39%)	3457 (15.35%)	11,005 (18.72%)	9185 (23.86%)	
**Ethnicity**						<0.001
Han	140,353 (91.80%)	31,386 (90.25%)	20,588 (91.44%)	53,545 (91.08%)	34,834 (90.49%)	
Minority	14,226 (9.20%)	3392 (9.75%)	1927 (8.56%)	5245 (8.92%)	3662 (9.51%)	
**Marital status**						<0.001
Unmarried	21,062 (13.63%)	5028 (14.46%)	3295 (14.63%)	8178 (13.91%)	4561 (11.85%)	
Married	129,163 (83.56%)	28,787 (82.77%)	18,669 (82.92%)	49,006 (83.36%)	32,701 (84.95%)	
Separated/divorced	4354 (2.82%)	963 (2.77%)	551 (2.45%)	1606 (2.73%)	1234 (3.21%)	
**Medical insurance**						<0.001
No	12,668 (8.20%)	3266 (9.39%)	1780 (7.91%)	4560 (7.76%)	3062 (7.95%)	
Yes	141,911 (91.81%)	31,512 (90.61%)	20,735 (92.09%)	54,230 (92.24%)	35,434 (92.05%)	
**Social insurance**						<0.001
No	76,534 (49.51%)	19,449 (55.92%)	11,852 (52.64%)	28,563 (48.58%)	16,670 (43.30%)	
Yes	78,045 (50.49%)	15,329 (44.08%)	10,663 (47.36%)	30,227 (51.42%)	21,826 (56.70%)	
**Hometown type**						<0.001
Rural	135,345 (87.56%)	31,953 (91.88%)	20,239 (89.89%)	51,282 (87.23%)	31,871 (82.79%)	
Suburban	11,068 (7.16%)	1771 (5.09%)	1372 (6.09%)	4304 (7.32%)	3621 (9.41%)	
Urban	8166 (5.28%)	1054 (3.03%)	904 (4.02%)	3204 (5.45%)	3004 (7.80%)	
**Migratory duration (year)**						<0.001
<10	118,616 (76.73%)	45,439 (79.31%)	16,440 (78.63%)	28,772 (75.95%)	27,965 (72.64%)	
≥10	35,963 (23.27%)	11,854 (20.69%)	4469 (21.37%)	9109 (24.05%)	10,531 (27.36%)	
**Migratory range**						<0.001
Interprovincial	74,870 (48.44%)	20,564 (59.13%)	11,146 (49.51%)	27,191 (46.25%)	15,969 (41.48%)	
Intercity in the province	51,680 (33.43%)	9747 (28.03%)	7455 (33.11%)	20,615 (35.07%)	13,863 (36.01%)	
Intercounty within the city	28,029 (18.13%)	4467 (12.84%)	3.914 (17.38%)	10,984 (18.68%)	8664 (22.51%)	
**Hypertension**						<0.001
No	146,884 (95.02%)	33,032 (94.98%)	21,540 (95.67%)	55,990 (95.24%)	36,332 (94.35%)	
Yes	7695 (4.98%)	1746 (5.02%)	975 (4.33%)	2800 (4.76%)	2174 (5.65%)	
**Diabetes**						<0.001
No	152,598 (98.72%)	34,388 (98.88%)	22,270 (98.91%)	58,055 (98.75%)	37.885 (98.41%)	
Yes	1.981 (1.28%)	390 (1.12%)	245 (1.09%)	735 (1.25%)	611 (1.59%)	
**Combined chronic diseases (hypertension and/or diabetes)**						<0.001
No	145,759 (94.29%)	32,797 (94.30%)	21,400 (95.05%)	55,568 (94.52%)	35,994 (93.50%)	
Yes	8820 (5.71%)	1981 (5.70%)	1115 (4.95%)	3222 (5.48%)	2502 (6.50%)	
**Social activity**						<0.001
No	85,115 (55.06%)	21,706 (62.41%)	13,042 (57.93%)	31,468 (53.53%)	18,899 (49,09%)	
Yes	69,464 (44.94%)	13,072 (37.59%)	9473 (42.07%)	27,322 (46.47%)	19,597 (50.91%)	

Note: ^1^ One-way ANOVA was used to determine the statistical significance of the social integration score. The statistical significance for categorical variables, including age, sex, income, education, ethnicity, marital status, medical insurance, hometown type, migration duration, migration range, hypertension, diabetes, combined chronic disease, social insurance, and participation in social activity were determined using the Pearson chi-square test. ^2^ Presented as median and interquartile range.

**Table 2:** Associations between social integration and hypertension, diabetes, and combined chronic diseases ^1^.

Characteristics	Hypertension		Diabetes		Combined Chronic Diseases	
	AOR (95% CI)	*p*-Value	AOR (95% CI)	*p*-Value	AOR (95% CI)	*p*-Value
**Social integration quartile**						
Q1	Ref		Ref		Ref	
Q2	0.89 (0.87, 0.90)	<0.001	0.98 (0.93, 1.03)	0.355	0.89 (0.88, 0.90)	<0.001
Q3	0.96 (0.95, 0.97)	<0.001	1.07 (1.04, 1.10)	<0.001	0.96 (0.94, 0.99)	0.003
Q4	1.08 (1.03, 1.13)	0.002	1.23 (1.15, 1.32)	<0.001	1.09 (1.03, 1.14)	<0.001
**Social insurance**						
No	Ref		Ref		Ref	
Yes	1.03 (0.92, 1.14)	0.650	1.09 (0.87, 1.37)	0.448	1.06 (0.93, 1.19)	0.388
**Social activity**						
No	Ref		Ref		Ref	
Yes	0.78 (0.77, 0.80)	<0.001	0.78 (0.71, 0.85)	<0.001	0.77 (0.76, 0.78)	<0.001
**Age**						
<35	Ref		Ref		Ref	
≥35	8.20 (7.55, 8.91)	<0.001	6.00 (4.98, 7.23)	<0.001	8.16 (7.36, 9.05)	<0.001
**Sex**						
Male	Ref		Ref		Ref	
Female	0.82 (0.73, 0.93)	<0.001	0.85 (0.79, 0.91)	<0.001	0.81 (0.72, 0.90)	<0.001
**Income**						
<CNY 6000	Ref		Ref		Ref	
≥CNY 6000	0.73 (0.67, 0.80)	<0.001	0.73 (0.64, 0.82)	<0.001	0.73 (0.66, 0.80)	<0.001
**Education**						
Middle school/Primary school/No formal school	Ref		Ref		Ref	
Secondary school	0.83 (0.80, 0.86)	<0.001	0.74 (0.60, 0.91)	0.005	0.80 (0.75, 0.85)	<0.001
College and above	0.64 (0.59, 0.69)	<0.001	0.65 (0.58, 0.73)	<0.001	0.62 (0.59, 0.65)	<0.001
**Ethnicity**						
Han	Ref		Ref		Ref	
Minority	1.00 (0.94, 1.06)	0.990	1.07 (1.02, 1.11)	0.003	1.04 (0.98, 1.10)	0.196
**Marital status**						
Unmarried	Ref		Ref		Ref	
Married	2.18 (1.77, 2.68)	<0.001	2.28 (1.88, 2.77)	<0.001	2.17 (1.73, 2.73)	<0.001
Separated/divorced	3.19 (2.90, 3.52)	<0.001	3.00 (2.66, 3.39)	<0.001	3.14 (2.89, 3.42)	<0.001
**Medical insurance**						
No	Ref		Ref		Ref	
Yes	1.13 (1.02, 1.26)	0.021	1.28 (0.89, 1.85)	0.185	1.15 (0.99, 1.34)	0.076
**Hometown type**						
Rural	Ref		Ref		Ref	
Suburban	1.56 (1.55, 1.58)	<0.001	2.26 (2.20, 2.31)	<0.001	1.64 (1.62, 1.65)	<0.001
Urban	1.98 (1.96, 1.99)	<0.001	2.99 (2.97, 3.02)	<0.001	2.11 (2.09, 2.14)	<0.001
**Migratory duration (year)**						
<10	Ref		Ref		Ref	
≥10	1.41 (1.29, 1.53)	<0.001	1.44 (1.28, 1.62)	<0.001	1.40 (1.28, 1.52)	<0.001
**Migratory range**						
Interprovincial	Ref		Ref		Ref	
Intercity in the province	1.04 (1.01, 1.07)	0.004	1.14 (0.97, 1.33)	0.114	1.05 (0.99, 1.11)	0.101
Intercounty within the city	1.20 (1.13, 1.28)	<0.001	1.30 (1.09, 1.54)	0.003	1.21 (1.13, 1.29)	<0.001

Note: ^1^ Combined chronic diseases: hypertension and/or diabetes. AOR: adjusted odds ratio; CI: confidence interval. Adjusted for age, sex, monthly income, education, ethnicity, marital status, medical insurance, hometown type (rural, suburban, urban), migration duration, migration range, social insurance, and participation in social activity.

## Data Availability

Restrictions apply to the availability of these data. Data were obtained from the National Earth System Science Data Center of China and are available at http://www.geodata.cn with the permission of the National Earth System Science Data Center.

## Appendix A

**Table A1:** The 8-item Likert scale for the measurement of social integration.

Items	Level of Agreement
A I like the city/place where I live now	1 = Totally disagree 2 = Disagree 3 = Agree 4 = Totally agree
B I pay attention to the changes in the city/place where I live now	1 = Totally disagree 2 = Disagree 3 = Agree 4 = Totally agree
C I am willing to be a part of local residents	1 = Totally disagree 2 = Disagree 3 = Agree 4 = Totally agree
D I think the locals are willing to accept me as one of them	1 = Totally disagree 2 = Disagree 3 = Agree 4 = Totally agree
E I feel that locals look down on outsiders	1 = Totally agree 2 = Agree 3 = Disagree 4 = Totally disagree
F It’s important for me to follow the customs of my hometown	1 = Totally agree 2 = Agree 3 = Disagree 4 = Totally disagree
G My hygiene habits are quite different from those of the local people	1 = Totally agree 2 = Agree 3 = Disagree 4 = Totally disagree
H I consider myself to be a local resident	1 = Totally disagree 2 = Disagree 3 = Agree 4 = Totally agree

**Table A2:** Measurement for covariates.

Covariates	Variable Description	Variable Type
Sex	0 = Male, 1 = Female	Binary
Age (year)	0 = Under 35 years old, 1 = 35 years old and above	Binary
Ethnicity	0 = Han, 1 = Others	Binary
Education	1 = No formal school/Primary school/Middle school, 2 = Secondary school, 3 = College and above	Categorical ordered
Marital status	1 = Unmarried, 2 = Currently married/living with spouse, 3 = Divorced/Widowed	Categorical
Monthly income (CNY)	0 = Less than CNY 6000, 1 = CNY 6000 and above	Binary
Medical insurance	0 = No, 1 = Yes	Binary
Location of hometown	1 = Rural, 2 = Suburban, 3 = Urban	Categorical
Duration of migration	0 = Less than 10 years, 1= 10 years and above	Binary
Migratory range	1 = Intercounty within the city, 2 = Intercity in the province, 3 = Intercounty within the city	Categorical
Participation in social activity	0 = No, 1= Yes	Binary
Social insurance	0 = No, 1 = Yes	Binary

**Table A3:** Association between social integration quartile and combined chronic diseases ^1^, disaggregated by age, sex, income, and migrant duration.

	Hypertention		Diabetes		Combined Chronic Diseases	
Characteristics	AOR (95% CI)	*p*-Value	AOR (95% CI)	*p*-Value	AOR (95% CI)	*p*-Value
**Disaggregated by age**						
Social integration quartile (<35 years)						
Q1	Ref		Ref		Ref	
Q2	0.93 (0.86, 1.01)	0.098	1.09 (1.00, 1.19)	0.036	0.90 (0.85, 0.95)	<0.001
Q3	0.78 (0.77, 0.79)	<0.001	0.70 (0.63, 0.78)	<0.001	0.74 (0.73, 0.75)	<0.001
Q4	0.75 (0.70, 0.82)	<0.001	0.82 (0.52, 1.30)	0.402	0.74 (0.69, 0.80)	<0.001
Social integration quartile (≥35 years)						
Q1	Ref		Ref		Ref	
Q2	0.88 (0.87, 0.89)	<0.001	0.96 (0.89, 1.02)	0.186	0.89 (0.87, 0.91)	<0.001
Q3	0.98 (0.97, 0.99)	<0.001	1.12 (1.10, 1.15)	<0.001	0.99 (0.97, 1.01)	0.485
Q4	1.12 (1.07, 1.17)	<0.001	1.29 (1.23, 1.35)	<0.001	1.13 (1.08, 1.18)	<0.001
**Disaggregated by sex**						
Social integration quartile (Male)						
Q1	Ref		Ref		Ref	
Q2	0.89 (0.86, 0.93)	<0.001	1.00 (0.90, 1.10)	0.947	0.89 (0.86, 0.93)	<0.001
Q3	0.92 (0.89, 0.95)	<0.001	1.05 (1.01, 1.10)	0.016	0.93 (0.88, 0.99)	0.016
Q4	1.06 (1.02, 1.11)	0.007	1.25 (1.17, 1.35)	<0.001	1.07 (1.03, 1.12)	0.002
Social integration quartile (Female)						
Q1	Ref		Ref		Ref	
Q2	0.87 (0.83, 0.92)	<0.001	0.95 (0.91, 1.00)	0.059	0.89 (0.86, 0.92)	<0.001
Q3	1.02 (0.98, 1.06)	0.412	1.08 (1.05, 1.14)	<0.001	1.01 (0.98, 1.05)	0.444
Q4	1.11 (1.01, 1.22)	0.025	1.21 (1.11, 1.30)	<0.001	1.12 (1.03, 1.22)	0.012
**Disaggregated by income**						
Social integration quartile (< CNY 6000)						
Q1	Ref		Ref		Ref	
Q2	0.91 (0.86, 0.98)	0.009	1.05 (0.95, 1.16)	0.330	1.12 (0.89, 1.41)	0.329
Q3	1.01 (0.99, 1.03)	0.316	1.10 (1.00, 1.20)	0.050	1.15 (1.07, 1.25)	<0.001
Q4	1.14 (1.10, 1.17)	<0.001	1.28 (1.22, 1.35)	<0.001	1.32 (1.23, 1.41)	<0.001
Social integration quartile (≥ CNY 6000)						
Q1	Ref		Ref		Ref	
Q2	0.84 (0.81, 0.88)	<0.001	0.89 (0.85, 0.91)	<0.001	0.97 (0.94, 1.02)	0.225
Q3	0.89 (0.85, 0.93)	<0.001	1.03 (0.91, 1.16)	0.652	1.13 (1.06, 1.20)	<0.001
Q4	1.00 (0.90, 1.12)	0.975	1.16 (1.01, 1.32)	0.036	1.49 (1.14, 1.97)	0.004
**Disaggregated by migratory duration (year)**						
Social integration quartile (<10 years)						
Q1	Ref		Ref		Ref	
Q2	0.92 (0.90, 0.94)	<0.001	0.98 (0.88, 1.10)	0.752	0.92 (0.85, 0.99)	0.019
Q3	1.00 (0.98, 1.02)	0.779	1.09 (0.98, 1.22)	0.108	0.98 (0.97, 0.99)	<0.001
Q4	1.20 (1.05, 1.38)	0.008	1.34 (1.07, 1.70)	0.012	1.15 (1.09, 1.21)	<0.001
Social integration quartile (≥10 years)						
Q1	Ref		Ref		Ref	
Q2	0.83 (0.76, 0.91)	<0.001	1.00 (0.79, 1.26)	0.999	0.85 (0.77, 0.92)	<0.001
Q3	0.92 (0.83, 1.02)	0.104	1.12 (1.02, 1.23)	0.021	0.93 (0.87, 1.00)	0.055
Q4	0.98 (0.91, 1.06)	0.677	1.27 (1.11, 1.47)	<0.001	0.99 (0.94, 1.04)	0.613

Note: ^1^ Combined chronic diseases: hypertension and/or diabetes. AOR: adjusted odds ratio; CI: confidence interval. Logistic regression analysis was used to examine the association between social integration and hypertension and/or diabetes, and disaggregated by each variable including age, sex, income, and migrant duration. Adjusted for age, sex, monthly income, education, ethnicity, marital status, medical insurance, hometown type (rural, suburban, urban), migration duration, migration range, social insurance, and participation in social activity.

## References

[1] Goodkind D., West L.A. (2002). China’s Floating Population: Definitions, Data and Recent Findings. Urban. Stud..

[2] Mou J., Griffiths S.M., Fong H.F., Dawes M.G. (2015). Defining migration and its health impact in China. Public Health.

[3] Zheng Y., Zhang X., Dai Q., Zhang X. (2020). To Float or Not to Float? Internal Migration of Skilled Laborers in China. Int. J. Env. Res. Public Health.

[4] Chai J.C.H., Karin Chai B. (1997). China’s floating population and its implications. Int. J. Soc. Econ..

[5] Communiqué of the Seventh National Population Census (No. 8) (2021). Office of the Leading Group of the State Council for the Seventh National Population Census, National Bureau of Statistics of China. https://www.stats.gov.cn/english/PressRelease/202105/t20210510_1817193.html.

[6] Why Isn’t China Considering Immigration Against Demographic Decline?. https://www.ispionline.it/en/institute.

[7] The Seventh National Population Census Bulletin [1] (No. 7)—Rural and Urban Population and Floating Population. http://www.stats.gov.cn/sj/zxfb/202302/t20230203_1901087.html.

[8] Migrant Workers and Their Children. https://clb.org.hk/en/content/migrant-workers-and-their-children.

[9] Wang J., Bai L., Xu X. (2024). Disparities in awareness and utilisation of National Essential Public Health Services between the floating population and the registered residents: A cross-sectional study in China. BMJ Open.

[10] Wu L., Li W., Wang S., Weihua G., Wang X. (2024). Research on the health status and influencing factors of the older adult floating population in Shanghai. Front. Public Health.

[11] Lin X., Mao X., Ai F., Yao W. (2023). Factors influencing utilization of communicable disease prevention and treatment education among the floating population: A cross-sectional study in China. BMC Public Health.

[12] Zhang J., Cai J., Huang Y., He Z., Tang G. (2021). China’s Floating Population’s Healthcare Utilization Choices and Influencing Factors. Chin. Gen. Pract..

[13] Hu X., Cook S., Salazar M.A. (2008). Internal migration and health in China. Lancet.

[14] Tang D., Bu T., Liu Y. (2022). Mobility-related inequality in healthcare utilization between floating and native populations and its influencing factors: Evidence from China. Int. Health.

[15] Hesketh T., Ye X.J., Li L., Wang H.M. (2008). Health status and access to health care of migrant workers in China. Public Health Rep..

[16] He W. (2023). Does the immediate reimbursement of medical insurance reduce the socioeconomic inequality in health among the floating population? Evidence from China. Int. J. Equity Health.

[17] Meng Y., Han J., Qin S. (2018). The Impact of Health Insurance Policy on the Health of the Senior Floating Population-Evidence from China. Int. J. Env. Res. Public Health.

[18] Cai X., Yang F., Bian Y. (2019). Gap analysis on hospitalized health service utilization in floating population covered by different medical insurances-----case study from Jiangsu Province, China. Int. J. Equity Health.

[19] Ma C., Zhang Y., Li Y., Wang Y., Jiang Y., Wang X., Ma S. (2020). Healthcare, Insurance, and Medical Expenditure of the Floating Population in Beijing, China. Front. Public Health.

[20] Ma C., Huo S., Chen H. (2021). Does integrated medical insurance system alleviate the difficulty of using cross-region health Care for the Migrant Parents in China-- evidence from the China migrants dynamic survey. BMC Health Serv. Res..

[21] Borhade A., Dey S. (2018). Do migrants have a mortality advantage?. Lancet.

[22] Moullan Y., Jusot F. (2014). Why is the ’healthy immigrant effect’ different between European countries?. Eur. J. Public Health.

[23] Záleská V., Brabcová I., Vacková J. (2014). Migration and its impact on mental and physical health: Social support and its main functions. Kontakt.

[24] Xiao Z., Xu S., Liu J. (2019). The Assessment of Social Integration of Urban Migrant Population: An Investigation Based on 50 Cities of Migration Destination. Popul. Res..

[25] Lin Y., Zhang Q., Chen W., Ling L. (2017). The social income inequality, social integration and health status of internal migrants in China. Int. J. Equity Health.

[26] Liang J., Shi Y., Osman M., Shrestha B., Wang P. (2020). The association between social integration and utilization of essential public health services among internal migrants in China: A multilevel logistic analysis. Int. J. Environ. Res. Public Health.

[27] Jing Z., Wang Y., Ding L., Tang X., Feng Y., Zhou C. (2019). Effect of social integration on the establishment of health records among elderly migrants in China: A nationwide cross-sectional study. BMJ Open.

[28] Zhang M., Shi Y., Zhou B., Huang Z., Zhao Z., Li C., Zhang X., Han G., Peng K., Li X. (2023). Prevalence, awareness, treatment, and control of hypertension in China, 2004–2018: Findings from six rounds of a national survey. BMJ.

[29] Wang L., Peng W., Zhao Z., Zhang M., Shi Z., Song Z., Zhang X., Li C., Huang Z., Sun X. (2021). Prevalence and Treatment of Diabetes in China, 2013-2018. Jama.

[30] Wang F., Wang W., Yin P., Liu Y., Liu J., Wang L., Qi J., You J., Lin L., Zhou M. (2022). Mortality and Years of Life Lost in Diabetes Mellitus and Its Subcategories in China and Its Provinces, 2005–2020. J. Diabetes Res..

[31] Bragg F., Holmes M.V., Iona A., Guo Y., Du H., Chen Y., Bian Z., Yang L., Herrington W., Bennett D. (2017). Association Between Diabetes and Cause-Specific Mortality in Rural and Urban Areas of China. JAMA.

[32] Lewington S., Lacey B., Clarke R., Guo Y., Kong X.L., Yang L., Chen Y., Bian Z., Chen J., Meng J. (2016). The Burden of Hypertension and Associated Risk for Cardiovascular Mortality in China. JAMA Intern. Med..

[33] Guan Y., Zhang M., Zhang X., Zhao Z., Huang Z., Li C., Xiao Q., Wang L. (2019). Association between sleep duration and hypertension of migrant workers in China: A national cross-sectional surveillance study. BMJ Open.

[34] Han K., Yao J., Yin X., Zhao M., Sun Q. (2017). Review on the prevalence of diabetes and risk factors and situation of disease management in floating population in China. Glob. Health Res. Policy.

[35] Wang Z., Wang Q., Qi J. (2021). Ten years of the China migrants dynamic survey: Retrospective and prospects. China Popul. Dev. Stud..

[36] Chen M., Guo S., Lu D. (2017). Characteristics and spatial patterns of floating population in the Beijing-Tianjin-Hebei urban agglomeration under the background of new urbanization. Prog. Geogr..

[37] How to Distinguish Between Resident Population and Floating Population. https://www.stats.gov.cn/zs/tjws/tjbz/202301/t20230101_1903796.html.

[38] Shen H., Xia L. (2023). Research on Social Integration Structure and Path of Floating Population Based on Structural Equation Model: Evidence from China. Soc. Indic. Res..

[39] Shen T., Lao X., Gu H.B. (2022). The Social Integration of Floating Population. Migration Patterns and Intentions of Floating Population in Transitional China: The Road for Urban Dream Chasers.

[40] Dunn J.R., Dyck I. (2000). Social determinants of health in Canada’s immigrant population: Results from the National Population Health Survey. Soc. Sci. Med..

[41] Pérez C.E. (2002). Health status and health behaviour among immigrants. Health Rep..

[42] Modesti P.A., Marzotti I., Calabrese M., Stefani L., Toncelli L., Modesti A., Galanti G., Boddi M. (2021). Gender differences in acculturation and cardiovascular disease risk-factor changes among Chinese immigrants in Italy: Evidence from a large population-based cohort. Int. J. Cardiol. Cardiovasc. Risk Prev..

[43] Wong S.S., Dixon L.B., Gilbride J.A., Kwan T.W., Stein R.A. (2013). Measures of acculturation are associated with cardiovascular disease risk factors, dietary intakes, and physical activity in older Chinese Americans in New York City. J. Immigr. Minor. Health.

[44] Commodore-Mensah Y., Ukonu N., Obisesan O., Aboagye J.K., Agyemang C., Reilly C.M., Dunbar S.B., Okosun I.S. (2016). Length of Residence in the United States is Associated With a Higher Prevalence of Cardiometabolic Risk Factors in Immigrants: A Contemporary Analysis of the National Health Interview Survey. J. Am. Heart Assoc..

[45] Divney A.A., Echeverria S.E., Thorpe L.E., Trinh-Shevrin C., Islam N.S. (2019). Hypertension Prevalence Jointly Influenced by Acculturation and Gender in US Immigrant Groups. Am. J. Hypertens..

[46] O’Brien M.J., Alos V.A., Davey A., Bueno A., Whitaker R.C. (2014). Acculturation and the prevalence of diabetes in US Latino Adults, National Health and Nutrition Examination Survey 2007–2010. Prev. Chronic Dis..

[47] Zhu N. (2002). The impacts of income gaps on migration decisions in China. China Econ. Rev..

[48] Huang L., Said R., Goh H.C., Cao Y. (2023). The Residential Environment and Health and Well-Being of Chinese Migrant Populations: A Systematic Review. Int. J. Env. Res. Public Health.

[49] MacLachlan I., Gong Y. (2022). China’s new age floating population: Talent workers and drifting elders. Cities.

[50] Sarich P.E., Ding D., Sitas F., Weber M.F. (2015). Co-occurrence of chronic disease lifestyle risk factors in middle-aged and older immigrants: A cross-sectional analysis of 264,102 Australians. Prev. Med..

[51] Lee S.K., Sobal J., Frongillo E.A. (2000). Acculturation and health in Korean Americans. Soc. Sci. Med..

[52] Marmot M.G., Syme S.L. (1976). Acculturation and coronary heart disease in Japanese-Americans. Am. J. Epidemiol..

[53] Bilecen B., Vacca R. (2021). The isolation paradox: A comparative study of social support and health across migrant generations in the U.S. Soc. Sci. Med..

[54] Treas J., Batalova J.B., Uhlenberg P. (2009). Immigrants and Aging. International Handbook of Population Aging.

[55] Harding B.N., Hawley C.N., Kalinowski J., Sims M., Muntner P., Young Mielcarek B.A., Heckbert S.R., Floyd J.S. (2022). Relationship between social support and incident hypertension in the Jackson Heart Study: A cohort study. BMJ Open.

[56] Nyaaba G.N., Stronks K., Meeks K., Beune E., Owusu-Dabo E., Addo J., de-Graft Aikins A., Mockenhaupt F., Bahendeka S., Klipstein-Grobusch K. (2019). Is social support associated with hypertension control among Ghanaian migrants in Europe and non-migrants in Ghana? The RODAM study. Intern. Emerg. Med..

[57] Zhou J., Zhu L., Zhang J. (2022). Social Integration and Health Among Young Migrants in China: Mediated by Social Mentality and Moderated by Gender. Front. Psychol..

[58] Martinovic B., Tubergen F.v., Maas I. (2009). Changes in immigrants’ social integration during the stay in the host country: The case of non-western immigrants in The Netherlands. Soc. Sci. Res..

[59] Lu C., Hu Y., Xie J., Fu Q., Leigh I., Governor S., Wang G. (2018). The Use of Mobile Health Applications to Improve Patient Experience: Cross-Sectional Study in Chinese Public Hospitals. JMIR mHealth uHealth.

[60] Xie Y., Guo Q., Meng Y. (2021). The health service use of aged rural-to-urban migrant workers in different types of cities in China. BMC Health Serv. Res..

[61] Liang Y., Guo M. (2014). Utilization of Health Services and Health-Related Quality of Life Research of Rural-to-Urban Migrants in China: A Cross-Sectional Analysis. Soc. Indic. Res..

[62] Qiu F., Kong Q., Fan D. (2023). Cumulative health disadvantages: An empirical study of the health and mobility of the first cohort of migrant workers in China. Front. Public Health.

[63] Guo H., AlSwayied G., Frost R., Rait G., Burns F. (2024). Barriers and facilitators to health-care access by older Chinese migrants in high-income countries: A mixed-methods systematic review. Ageing Soc..

[64] Zhang N. (2023). Aging, Migration, and Grandparenting in Post–One Child Policy China. Innov. Aging.

[65] Spruill T.M. (2010). Chronic psychosocial stress and hypertension. Curr. Hypertens. Rep..

[66] Surwit R.S., Schneider M.S., Feinglos M.N. (1992). Stress and diabetes mellitus. Diabetes Care.

[67] Kalmijn M. (2018). Contact and conflict between adult children and their parents in immigrant families: Is integration problematic for family relationships?. J. Ethn. Migr. Stud..

[68] Anastasiadou A., Kim J., Sanlitürk A.E., de Valk H., Zagheni E.B. (2023). Sex-and Gender-Based Differences in the Migration Process: A Systematic Literature Review.

[69] Boyd M., Grieco E. (2003). Women and migration: Incorporating gender into international migration theory. Migr. Inf. Source.

[70] Read J.G., Reynolds M.M. (2012). Gender differences in immigrant health: The case of Mexican and Middle Eastern immigrants. J. Health Soc. Behav..

[71] Gorman B.K., Read J.G., Krueger P.M. (2010). Gender, acculturation, and health among Mexican Americans. J. Health Soc. Behav..

[72] OECD (2019). Settling In 2018: Indicators of Immigrant Integration. Gender Differences in Immigrant Integration.

[73] Bach A. (2009). Immigration, integration and asylum policies from a gender perspective. European Social Watch Report.

[74] Earl E.M. (2019). Gender and Social Acceptance of Immigrants in a New Destination Site.

[75] Ten Kate R.L.F., Fokkema T., van Tilburg T.G. (2024). Gender Differences in Social Embeddedness Determinants of Loneliness Among Moroccan and Turkish Older Migrants. J. Gerontol. B Psychol. Sci. Soc. Sci..

[76] Avenarius C.B. (2012). Immigrant Networks in New Urban Spaces: Gender and Social Integration. Int. Migr..

[77] Curran S.R., Saguy A.C. (2001). Migration and cultural change: A role for gender and social networks?. J. Int. Women’s Stud..

[78] Brabete A.C.B., Sánchez-López M.P., Limiñana-Gras R.M. (2017). Examining Migrants’ Health From a Gender Perspective. The Psychology of Gender and Health.

[79] World Bank, Development Research Center of the State Council, the People’s Republic of China (2022). Four Decades of Poverty Reduction in China: Drivers, Insights for the World, and the Way Ahead.

[80] Swider S. (2014). Building China: Precarious employment among migrant construction workers. Work. Employ. Soc..

[81] Mou J., Griffiths S.M., Fong H., Dawes M.G. (2013). Health of China’s rural-urban migrants and their families: A review of literature from 2000 to 2012. Br. Med. Bull..

[82] Wang L., Zhang Z., Chang Y., Wang X., Hou M., Wei J., Ling W., Zhu H. (2011). Comparison of dietary habits between migrant and local adolescents in Shenzhen, China. Asia Pac. J. Clin. Nutr..

[83] Rosella L.C., Wodchis W.P., Yao Z., Sutradhar R., Ng R. (2020). Smoking, drinking, diet and physical activity—Modifiable lifestyle risk factors and their associations with age to first chronic disease. Int. J. Epidemiol..

[84] Tan S., Li Y., Song Y., Luo X., Zhou M., Zhang L., Kuang B. (2015). Influence factors on settlement intention for floating population in urban area: A China study. Qual. Quant..

[85] Hagos R.M., Hamilton T.G. (2024). Beyond Acculturation: Health and Immigrants’ Social Integration in the United States. J. Health Soc. Behav..

[86] Moran A., Diezroux A., Jackson S., Kramer H., Manolio T., Shrager S., Shea S. (2007). Acculturation Is Associated With Hypertension in a Multiethnic Sample. Am. J. Hypertens..

[87] Roura M. (2017). Unravelling migrants’ health paradoxes: A transdisciplinary research agenda. J. Epidemiol. Community Health.

[88] Alidu L., Grunfeld E.A. (2018). A systematic review of acculturation, obesity and health behaviours among migrants to high-income countries. Psychol. Health.

[89] OECD/European Commission (2023). Indicators of Immigrant Integration 2023.

[90] European Commission (2013). Using EU Indicators of Immigrant Integration. Brussels. https://migrant-integration.ec.europa.eu/library-document/using-eu-indicators-immigrant-integration_en.

[91] Substance Abuse and Mental Health Services Administration (US) (2014). Appendix B: Instruments To Measure Identity and Acculturation. Treatment Improvement Protocol (TIP).

[92] Kang X., Du M., Wang S., Du H. (2022). Exploring the Effect of Health on Migrants’ Social Integration in China. Int. J. Environ. Res. Public Health.

